# The burden of hospital admissions for skeletal dysplasias in Sri Lanka: a population-based study

**DOI:** 10.1186/s13023-023-02884-2

**Published:** 2023-09-08

**Authors:** Yasas D. Kolambage, Yasaswi N. Walpita, Udari A. Liyanage, Buddika M.K.D.R. Dayaratne, Vajira H.W. Dissanayake

**Affiliations:** 1 Department of Anatomy, Faculty of Medicine, Sabaragamuwa University of Sri Lanka, Ratnapura, Sri Lanka; 2 Department of Community Medicine, Faculty of Medicine, University of Colombo, Colombo, Sri Lanka; 3 Department of Anatomy, Genetics and Biomedical Informatics, Faculty of Medicine, University of Colombo, Colombo, Sri Lanka; 4 Medical Statistics Unit, Ministry of Health, Colombo, Sri Lanka

**Keywords:** Skeletal dysplasia, Osteochondrodysplasia, Hospital admission, Crude admission rate, Burden of Disease, Population-based study, Sri Lanka, Lower-middle income country.

## Abstract

**Background:**

Skeletal dysplasias are a diverse group of rare disorders in the chondro-osseous tissue that can have a significant impact on patient’s functionality. The worldwide prevalence of skeletal dysplasias at birth is approximately 1:5000 births. To date, disease burden and trends of skeletal dysplasias in the Sri Lankan population have not been described in any epidemiological study. Our aim was to evaluate the burden and the current trends in hospital admissions for skeletal dysplasias in the Sri Lankan population. A retrospective evaluation of hospital admissions for skeletal dysplasia during 2017–2020 was performed using population-based data from the eIMMR database which covers government hospitals in the entire country. The trends in hospital admissions for skeletal dysplasias by calendar year, age, and types of skeletal dysplasia were described using appropriate summary statistics.

**Results:**

Respective crude admission rates of skeletal dysplasias in the years 2017, 2018, 2019 and 2020 were 5.2, 8.1, 8.0, and 6.5 per million population. A female predominance (1.4:1) was noted during the studied period. Of all reported cases the majority (n = 268; 44.2%) were children less than 4 years. Each year, 0–4 years age group represented 40–47% of the total hospital admissions. More than half of the cases were reported from Colombo (28.1%) and Kandy (25.4%) districts combined. 60% of cases were diagnosed as osteogenesis imperfecta (OI). Rising trends were observed in the hospital admissions for osteogenesis imperfecta, achondroplasia and osteopetrosis, while other skeletal dysplasia types collectively showed a relatively stable trend.

**Conclusion:**

This preliminary study revealed a female predominance of skeletal dysplasias and a relatively high admission rate of osteogenesis imperfecta in the Sri Lankan population. A distinct trend was not visible in the studied years probably due to the impact on hospital services due to COVID- Pandemic. Future research on the healthcare burden on families affected by skeletal dysplasia is required to better understand the overall cost of care and identify therapies that reduce admission rates. This study highlights the value of analysing population-based data on rare diseases to improve healthcare in low-resource countries.

**Supplementary Information:**

The online version contains supplementary material available at 10.1186/s13023-023-02884-2.

## Background

Skeletal dysplasias or osteochondrodysplasias are a group of heritable disorders that have generalized developmental abnormalities in bone and cartilage, causing disability and disfiguring of variable extent [1]. Nosology and classification of genetic skeletal disorders, which had its latest revision in 2023, contains 771 disease entries listed under 41 different groups, arising from pathogenic variants in 552 different genes [2]. They affect one in every 3000 to 5000 births worldwide [3, 4]. The degree of severity could vary from mild short stature to perinatal lethality. They typically show symptoms after birth, particularly in the first several years of life. Patients have to get admitted to a hospital for the initial diagnosis and subsequent treatment and may require continued medical care for the rest of their lives. However, there is little information regarding the rate of hospitalization due to skeletal dysplasias globally.

In the Sri Lankan context, integration of genomic medicine into the daily clinical practice has allowed accurate diagnosis of rare disorders leading to enhanced patient care [5]. Nevertheless, the majority of skeletal dysplasias are still diagnosed by clinical and radiographic evidence alone. The authors were able to identify very few research focusing on skeletal dysplasias in the Sri Lankan population to date, and none of them were substantial studies [6–10]. There were no epidemiological studies describing the disease burden and trends in the Sri Lankan population, which is essential for effective nationwide service planning.

The electronic Indoor Morbidity and Mortality Reporting (eIMMR) System is a real-time healthcare information gathering system of the Ministry of Health, Sri Lanka. It gathers morbidity and mortality data by admissions from almost all government hospitals in the entire country, including data related to all diagnosed cases of skeletal dysplasias [11]. Around 95% of the country’s inward care is provided by the government, with the remaining portion being covered by the private sector [12]. During the study period, the eIMMR system demonstrated remarkable data coverage, capturing over 90% of the yearly hospitalization episodes in Sri Lanka, reflecting its capability to gather medical data at a national level (see Additional file 1). Therefore, eIMMR is a valuable resource which enables the analysis of disease trends by admission rates, bridging the population-based knowledge gap between primary and secondary prevention of skeletal dysplasias in Sri Lanka.

## Methods

The aim of this study was to calculate the crude rate of admissions for skeletal dysplasias to Sri Lankan government hospitals during 2017–2020 and to evaluate the current trends in hospital admissions in the Sri Lankan population.

### Study design and data sources

This was a retrospective cross-sectional study involving secondary data analysis. Records of all patients admitted to any government hospital, who received the diagnosis and treatment for skeletal dysplasias between 01.01.2017 and 31.12.2020, were retrieved from the electronic Indoor Morbidity and Mortality Record (eIMMR) System on 12 September 2022 [13]. In the eIMMR system, hospital admissions are coded with diagnoses using the International Classification of Diseases, 10th revision classification system (ICD-10) [14]. Inclusion in the study was based on appropriate ICD codes for skeletal dysplasias, as outlined in Additional file 2. Any records with disorders not covered by the specified ICD codes were excluded from the study analysis. Only cases with a primary diagnosis of skeletal dysplasia were included, and cases with a background of skeletal dysplasia but having different primary diagnoses were excluded from the study. This study relied solely on the aggregated information available in the eIMMR database and did not involve individual patient assessments. The categorical data including hospital admissions by gender, geographical location and specific disease entities were summarized as percentages.

### Statistical methods

The socio-demographic factors and reported specific skeletal dysplasia types were mainly analysed descriptively. Yearly crude admission rates of skeletal dysplasias per one million population with corresponding 95% confidence intervals were calculated using the estimated mid-year population of Sri Lanka, published by the Registrar General’s Department, Sri Lanka [15]. Similar methods were used to determine age group specific admission rates of skeletal dysplasias for each year under consideration. Patients were categorized into five age groups (preschool 0–4, young children 5–9, older children 10–14, adolescents 15–19 and adults more than 20 years). Population estimates for different age groups were calculated from the age distribution of Census of Population and Housing 2012.

### Ethical considerations

There was no direct patient involvement in this study as we evaluated data from a population database. Ethical approval for responsible management of information was obtained from the Ethics Review Committee of the Faculty of Medicine, University of Colombo (EC-19-138).

## Results

The estimated mid-year population of Sri Lanka has increased from 21,444,000 in 2017 to 21,919,000 in 2020. During this period, 606 admissions were recorded with a primary diagnosis of skeletal dysplasias in the eIMMR dataset (Table 1). Among these, 352 were female and 254 were males. The female to male ratio was 1.4: 1. More than 50% of the admissions were reported from Colombo and Kandy districts alone. Different subtypes of skeletal dysplasias were observed (Table 2). These included 367 (60.6%) osteogenesis imperfecta patients, 46 (7.6%) achondroplasia patients, 43 (7.1%) osteopetrosis patients and 150 (24.8%) patients with other skeletal dysplasias (The complete list of skeletal dysplasias is available in Additional file 2).

**Table 1:** Socio-demographic characteristics of the hospital admissions with a primary diagnosis of a skeletal dysplasia in Sri Lanka during 2017–2020

Characteristics	2017		2018		2019		2020		Total	
	No.	%	No.	%	No.	%	No.	%	No.	%
Gender										
Male	48	42.9	86	48.9	59	33.7	61	42.7	254	41.9
Female	64	57.1	90	51.1	116	66.3	82	57.3	352	58.1
**Total**	**112**	**100**	**176**	**100**	**175**	**100**	**143**	**100**	**606**	**100**
**District**										
Colombo	26	23.2	47	26.7	52	29.7	45	31.5	170	28.1
Kandy	48	42.9	34	19.3	42	24.0	30	21.0	154	25.4
Anuradhapura	7	6.3	15	8.5	28	16.0	17	11.9	67	11.1
Galle	5	4.5	19	10.8	7	4.0	11	7.7	42	6.9
Batticaloa	4	3.6	19	10.8	19	10.9	10	7.0	52	8.6
Other districts	22	19.6	42	23.9	27	15.4	30	21.0	121	20.0
**Total**	**112**	**100**	**176**	**100**	**175**	**100**	**143**	**100**	**606**	**100**

Percentage (%) values were rounded up to the first decimal point for presentation purposes. Hence, total might not add up to 100.0%

**Table 2:** Types of skeletal dysplasias recorded in hospital admissions in Sri Lanka during 2017–2020

Type of SD (ICD code)	2017		2018		2019		2020		Total	
	No.	%	No.	%	No.	%	No.	%	No.	%
OI (Q78.0)	67	58.9	112	63.6	101	57.7	87	60.8	367	60.6
ACH (Q77.4)	5	4.5	10	5.7	17	9.7	14	9.8	46	7.6
OPT (Q78.2)	4	3.6	8	4.5	19	10.9	12	8.4	43	7.1
Other SD^a^	36	33.0	46	26.1	38	21.7	30	21.0	150	24.8
**Total**	**112**	**100**	**176**	**100**	**175**	**100**	**143**	**100**	**606**	**100**

Percentage (%) values were rounded up to the first decimal point for presentation purposes. Hence, total might not add up to 100.0%. OI, osteogenesis imperfecta; ACH, Achondroplasia; SD, skeletal dysplasias

^a^ICD codes for other skeletal dysplasias are listed in Additional file 2

As Table 3 indicated, the crude admission rates of Skeletal dysplasias in the years 2017, 2018, 2019 and 2020 were 5.2, 8.1, 8.0 and 6.5 per million population respectively. Each year, 0–4 years age group represented 40–47% of the total hospital admissions, whereas less than 6% were reported from adults more than 20 years of age. The number of admissions in the age groups of 5 to 9 and 10 to 14 were roughly equal each year, having a crude rate between 14 and 22 per million population.

**Table 3:** Age specific crude admission rates of skeletal dysplasias per 1,000,000 population per year in Sri Lanka during 2017–2020

	2017			2018			2019			2020			Total		
Age group	N (% total)	Population^a^ (in 1000s)	Crude Rate (95% CI)	N (% total)	Population^a^ (in 1000s)	Crude Rate (95% CI)	N (% total)	Population^a^ (in 1000s)	Crude Rate (95% CI)	N (% total)	Population^a^ (in 1000s)	Crude Rate (95% CI)	N (% total)	Average Population (in 1000s)	Crude Rate (95% CI)
**0–4**	45	1839	24.5	76	1859	40.9	80	1871	42.8	67	1881	35.6	268	1862.5	143.9
	(40.2%)		(17.3–31.6)	(43.2%)		(31.7–50.1)	(45.7%)		(33.4–52.1)	(46.9%)		(27.1–44.1)	(44.2%)		(126.7-161.1)
**5–9**	28	1843	15.2	40	1863	21.5	42	1874	22.4	28	1885	14.9	138	1866.25	73.9
	(25%)		(9.6–20.8)	(22.7%)		(14.8–28.1)	(24%)		(15.6–29.2)	(19.6%)		(9.4–20.4)	(22.8%)		(61.6–86.3)
**10–14**	25	1729	14.5	40	1748	22.9	39	1759	22.2	27	1768	15.3	131	1751	74.8
	(22.3%)		(8.8–20.1)	(22.7%)		(15.8–30.0)	(22.3%)		(15.2–29.1)	(18.9%)		(9.5–21.0)	(21.6%)		(62.0-87.6)
**15–19**	12	1733	6.9	10	1752	5.7	12	1763	6.8	14	1773	7.9	48	1755.25	27.3
	(10.7%)		(3.0-10.8)	(5.7%)		(2.2–9.3)	(6.9%)		(3.0-10.7)	(9.8%)		(3.8–12.0)	(7.9%)		(19.6–35.1)
**20+**	2	14,300	0.1	10	14,448	0.7	2	14,536	0.1	7	14,612	0.5	21	14,474	1.5
	(1.8%)		(N/A^b^)	(5.7%)		(0.3–1.1)	(1.1%)		(N/A^b^)	(4.9%)		(N/A^b^)	(3.5%)		(0.8–2.1)
**All ages**	**112**	**21,444**	**5.2**	**176**	**21,670**	**8.1**	**175**	**21,803**	**8.0**	**143**	**21,919**	**6.5**	**606**	**21,709**	**27.9**
			**(4.3–6.2)**			**(6.9–9.3)**			**(6.8–9.2)**			**(5.5–7.6)**			**(25.7–30.1)**

Percentage (%) values were rounded up to the first decimal point for presentation purposes. Hence, total might not add up to 100.0%. N, number of hospital admissions; CI, confidence interval; N/A, not applicable

^a^Mid-year population data retrieved from Registrar General’s Department, Sri Lanka [15]. Age-specific population estimates have been calculated using the age distribution of Census of Population and Housing 2012

^b^Confidence intervals should not be calculated and used for crude rates based on less than 10 events, as they are not reliable

**Fig. 1:**
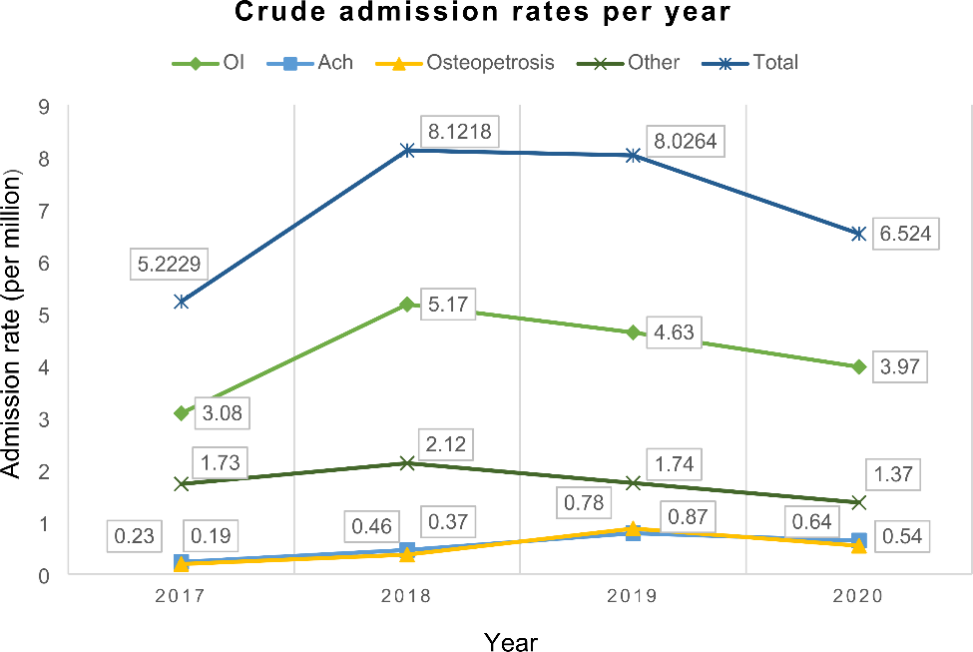
Trends of crude admission rates for different skeletal dysplasia types in Sri Lanka between 2017 and 2020. Abbreviations: Ach, achondroplasia; OI, osteogenesis imperfecta

When comparing the trend of crude admission rates of specific types of skeletal dysplasias (Fig. 1), a high rate of admissions for osteogenesis imperfecta was observed during the entire study period. It peaked in 2018 with a rate of 5.17 per million population, followed by a gradual decline. From 2017 to 2019, the percentage change in crude admission rates in OI was 50.3%, whereas Achondroplasia and Osteopetrosis had a considerably greater change (239.1% and 357.9%), respectively (Table 4). However, the rate of other skeletal dysplasias was minimally changed (0.5%).

**Table 4:** Percentage change in the crude admission rates for specific types of skeletal dysplasias from 2017 to 2019

Disease type	Crude rate in 2017 (A)	Crude rate in 2019 (B)	Percentage change^a^ from 2017 to 2019^b^
Osteogenesis imperfecta	3.08	4.63	50.3%
Achondroplasia	0.23	0.78	239.1%
Osteopetrosis	0.19	0.87	357.9%
Other skeletal dysplasias	1.73	1.74	0.5%
**Total**	**5.22**	**8.03**	**53.9%**

^a^Percentage change = (B-A)/A×100%,

^b^The influence of the COVID-19 pandemic on data prevented consideration of the crude rate in 2020

A detailed breakdown of the number of admissions for each disease category in each age category is shown in Table 5. In general, the number of hospitalizations declined with age, with the exception of admissions for osteopetrosis. The highest number of osteopetrosis patients was found in the age category of 10–14 years.

**Table 5:** Number of skeletal dysplasia patients admitted to government hospitals in Sri Lanka between 2017 and 2020 according to the age of patient

Disease category	Age 0–4	Age 5–9	Age 10–14	Age 15–19	Age 20+	Total (%)
Q77 Osteochondrodysplasia with defects of growth of tubular bones and spine	0	0	1	0	0	1 (0.2%)
Q77.1 Thanatophoric short stature	2	0	0	0	0	2 (0.3%)
Q77.2 Short rib syndrome	7	0	0	1	0	8 (1.3%)
Q77.3 Chondrodysplasia punctata	3	1	0	0	0	4 (0.7%)
Q77.4 Achondroplasia	32	6	3	2	3	46 (7.6%)
Q77.7 Spondyloepiphyseal dysplasia	0	0	1	0	1	2 (0.3%)
Q78 Other osteochondrodysplasias	29	21	12	12	1	75 (12.4%)
Q78.0 Osteogenesis imperfecta	152	101	80	28	6	367 (60.6%)
Q78.1 Polyostotic fibrous dysplasia	9	1	4	1	2	17 (2.8%)
Q78.2 Osteopetrosis	13	1	27	0	2	43 (7.1%)
Q78.3 Progressive diaphyseal dysplasia	2	0	0	0	0	2 (0.3%)
Q78.4 Enchondromatosis	4	1	0	0	0	5 (0.8%)
Q78.5 Metaphyseal dysplasia	0	0	0	0	1	1 (0.2%)
Q78.6 Multiple congenital exostoses	4	1	0	2	0	7 (1.2%)
Q78.8 Other specified osteochondrodysplasias	6	2	2	0	2	12 (2.0%)
Q78.9 Osteochondrodysplasia, unspecified	5	3	1	2	3	14 (2.3%)
**Total** **(%)**	**268** **(44.2%)**	**138** **(22.8%)**	**131** **(21.6%)**	**48** **(7.9%)**	**21** **(3.5%)**	**606** **(100%)**

Percentage (%) values were rounded up to the first decimal point for presentation purposes. Hence, total might not add up to 100.0%

## Discussion

To the best of our knowledge, this study represents the first-ever examination of trends in hospital admissions for skeletal dysplasias group using population-based data. Using data from the eIMMR system, we were able to identify 606 admissions for skeletal dysplasias between 2017 and 2020. Utilizing the specific eIMMR dataset proved beneficial, as it recorded 25,748,103 (95%) out of the total 27,289,524 hospitalization episodes in Sri Lanka during this period (see Additional file 1). However, the deployment of eIMMR is still not complete, because of certain infrastructure gaps that are particularly evident in remote hospitals. According to a recent study conducted in the eastern province of Sri Lanka, data input facilities for the eIMMR system have been introduced in approximately 80% of hospitals [16]. Moreover, it is important to highlight that the eIMMR data could not be used to calculate the incidence rates of skeletal dysplasias, because multiple entries from a single patient may exist due to the lack of a unique patient identifying mechanism in the system [11]. Therefore, this preliminary study demonstrated trends in crude admission rates of overall and specific types of skeletal dysplasias. However, the hospital admission rates are also an important parameter in analysing the burden of skeletal dysplasia on health systems and for resource mobilization.

Over the course of the study period, yearly crude admission rates of skeletal dysplasias were at their highest recorded level in 2018, and the trend shows a steady drop afterward. We were unable to compare this trend to those of previous studies since there were limited worldwide data on the trends of hospital admissions for skeletal dysplasias. The total yearly number of hospital admissions for skeletal dysplasias decreased by 18.3% from 175 to 2019 to 143 in 2020. During the lockdown period brought on by the coronavirus pandemic, it has been reported that hospital admissions in Sri Lanka plummeted [17]. This could explain the considerable decrease in the number of patients hospitalized with skeletal dysplasias in 2020. On that account, we opted out post-COVID-19 data from our trend analysis and considered it as an outlier.

According to this study, the ratio of female to male hospital admissions was 1.4:1. The observed female predominance in hospital admissions for skeletal dysplasia may be attributed to a higher proportion of females being diagnosed with the condition, potentially due to sex-specific differences in disease presentation or identification. A similar female predominance was observed in a cohort of skeletal dysplasias children with short stature [18] and in several other studies that investigated birth prevalence rates of skeletal dysplasias [3, 19, 20]. This disparity might be explained by the larger proportion of female patients admitted to hospitals. Since the majority of skeletal dysplasias are autosomal disorders, theoretically an equal gender distribution is anticipated. It would be interesting to study the in-depth basis behind these observations in future epidemiological studies.

When analysing the health burden of specific disease entities, we found that admissions for osteogenesis imperfecta (OI) were the most common and accounted for the highest crude admission rate across the study period. This finding could be attributed to either a high readmission rate or a high prevalence rate of OI. Storoni et al. revealed that people with OI are hospitalized 2.9 times more frequently compared to the general population [21]. Another study conducted in the United Kingdom reported that OI patients had higher hospitalizations per year on average compared to other patients [22]. Patients with OI experience a variety of medical complications that may require a variety of surgical interventions over their lifetimes. Thus, recurring fracture episodes in OI patients may have resulted in frequent non-elective readmissions. On the other hand, OI has a high prevalence rate, which could lead to a high crude admission rate. In terms of skeletal dysplasia birth prevalence studies, larger multicentric and population-based studies reported a higher rate of OI than achondroplasia [23, 24], while some studies demonstrated comparable rates in both conditions [3, 20]. As both factors may have contributed to our finding, further research is crucial in determining whether better care is needed to reduce avoidable hospital readmissions in Sri Lanka.

In our study, the highest crude admission rate for skeletal dysplasias was noted in the 0–4 years age group. The age of diagnosis strongly associates with the peak age of hospital admissions for skeletal dysplasias, since patients must be hospitalized several times to explore the diagnosis. As skeletal dysplasias are genetic disorders that affect foetal and early childhood skeletal development, they can manifest either prenatally or in the first few months of life [25]. This could explain the higher number of preschool (0–4 years) admissions. Likewise, the age of diagnosis can vary depending on the specific type of skeletal dysplasia, with most types becoming apparent earlier in life, while others may not be diagnosed until later in childhood or even adulthood [1]. We observed that the majority of osteopetrosis cases were hospitalized in late childhood, in the age group of 10–14 years. The age of onset of osteopetrosis can vary according to three distinct clinical forms of the disease, with cases presenting in infancy (malignant, autosomal recessive), in childhood (intermediate, autosomal recessive), or in adulthood (Albers-Schönberg disease, autosomal dominant) [26]. One possible explanation could be the presence of specific genetic variants that are more commonly found in the Sri Lankan population, leading to a higher incidence of marble bone disease. Additionally, limited access to adequate medical care and diagnostic facilities may also play a role in the age of onset of osteopetrosis. The higher admission rate of childhood cases in Sri Lanka highlights the importance of early diagnosis and treatment for osteopetrosis, particularly in resource-limited settings.

Our analysis found a 54% rise in all skeletal dysplasias hospitalizations from 2017 to 2019. Firstly, it is possible that relatively low rates in the early years resulted from underreporting due to a lack of adaption to the eIMMR system. Secondly, this could be attributed to improved diagnostic ability in relation to these specific disorders. The implementation of genomic medicine into routine clinical practice may have had an effect on the number of diagnosed rare and complex disorders such as skeletal dysplasias, resulting in an increase in hospital admissions [5]. Nevertheless, osteogenesis imperfecta, achondroplasia, and osteopetrosis admissions have increased exponentially compared to other types of skeletal dysplasias during the given timeframe. It is important to note that genomic medicine is rarely offered to diagnose these three disorders in developing countries because clinical and radiological evidence is usually sufficient [27]. We might therefore conclude that the first explanation must have had a greater impact than the latter.

One of the main limitations of this study is that a few skeletal dysplasia types were excluded since they were not categorized under osteochondrodysplasias in the ICD-10 (Q77 and Q78). Because of this limitation, the burden of skeletal dysplasia hospital admissions in Sri Lanka may have been underestimated. Further the variable rates of hospital admissions required by different conditions depending on the management protocols of each skeletal dysplasia may have led to an over or underestimation of certain skeletal dysplasias. This limitation has arisen from the fact that the eIMMR in Sri Lanka is not linked to a unique patient ID. The other limitation of the study is that the eIMMR database does not provide specific details about the diagnostic process. This lack of detailed information on how the diagnosis was initially made can potentially limit the depth of the study’s analysis and understanding of the diagnostic procedures used for skeletal dysplasias.

## Conclusion

Skeletal dysplasias present a diagnostic challenge due to their heterogeneous nature, resulting in a significant burden on healthcare systems. This study provides valuable insight into the burden of skeletal dysplasia hospital admissions in Sri Lanka, revealing a female predominance and a high admission rate of osteogenesis imperfecta. While these findings are a crucial first step, further research is necessary to fully understand the scope of this important health problem. The study also highlights the value of using existing population-based data to improve healthcare for rare diseases in low-resource countries.

### Electronic supplementary material

Below are the links to the electronic supplementary material.

**Supplementary Table S1** Number of eIMMR records, total hospitalization episodes and the eIMMR coverage in Sri Lanka during 2017-2020.

**Supplementary Table S2** ICD-10 codes used for skeletal dysplasias.

## Data Availability

The original eIMMR data supporting this study’s findings are not publicly available due to policy constraints and are stored in controlled access data storage at the Medical Statistics Unit, Ministry of Health, Sri Lanka. However, the secondary datasets derived from the original data can be obtained from the corresponding author upon reasonable request through Harvard Dataverse with the identifier 10.7910/DVN/9WRBL7. Additionally, the midyear population data in Table 3 are accessible at the following URL: http://www.statistics.gov.lk/Resource/en/Population/Vital_Statistics/Mid-year_population_by_age_group.pdf.
