# Supplementary material for: The burden of hospital admissions for skeletal dysplasias in Sri Lanka: a population-based study
Source: Orphanet J Rare Dis. 2023 Sep 8;18:279. doi: 10.1186/s13023-023-02884-2 (PMC10485930; doi:10.1186/s13023-023-02884-2)
Supplement: Supplementary file 1 — Supplementary Table S1 Number of eIMMR records, total hospitalization episodes and the eIMMR coverage in Sri Lanka during 2017-2020. [file 13023_2023_2884_MOESM1_ESM.docx]

**Supplementary Table S1** Number of eIMMR records, total hospitalization episodes and the eIMMR coverage in Sri Lanka during 2017-2020.

| **Year** | **Number of eIMMR records** | **Total hospitalization episodes^a^** | **eIMMR coverage^b^ (%)** |
| --- | --- | --- | --- |
| 2017 | 6219317 | 6910249 | 90.0 |
| 2018 | 6803960 | 7116268 | 95.6 |
| 2019 | 7124421 | 7477860 | 95.3 |
| 2020 | 5772504 | 5785147^c^ | 99.8 |
| **Total** | **25748103** | **27289524** | **95.0** |

Source: Medical Statistics Unit, Ministry of Health, Sri Lanka.
^a^The Medical Statistics Unit collects hospital admission data through traditional means, separate from eIMMR, and publishes it in the Annual Health Bulletin [1]
^b^eIMMR Coverage is determined by dividing the number of eIMMR records by the total hospital admission episodes and expressing it as a percentage.
^c^The COVID-19 pandemic had an impact on the data entry in several hospitals.

**References**

1. Medical Statistics Unit. Annual Health Bulletin 2020. Colombo: Medical Statistics Unit, Ministry of Health, Sri Lanka. 2023.
