# Supplementary material for: The burden of hospital admissions for skeletal dysplasias in Sri Lanka: a population-based study
Source: Orphanet J Rare Dis. 2023 Sep 8;18:279. doi: 10.1186/s13023-023-02884-2 (PMC10485930; doi:10.1186/s13023-023-02884-2)
Supplement: Supplementary file 2 — Supplementary Table S2 ICD-10 codes used for skeletal dysplasias. [file 13023_2023_2884_MOESM2_ESM.docx]

**Supplementary Table S2** ICD-10 codes used for skeletal dysplasias [1]

| **Code** | **Diseases** |
| --- | --- |
| **Q77** | **Osteochondrodysplasia with defects of growth of tubular bones and spine** |
| Q77.0 | Achondrogenesis  Hypochondrogenesis |
| Q77.1 | Thanatophoric short stature |
| Q77.2 | Short rib syndrome  Asphyxiating thoracic dysplasia [Jeune] |
| Q77.3 | Chondrodysplasia punctata |
| Q77.4 | Achondroplasia  Hypochondroplasia  Osteosclerosis congenita |
| Q77.5 | Dystrophic dysplasia |
| Q77.6 | Chondroectodermal dysplasia  Ellis-van Creveld syndrome |
| Q77.7 | Spondyloepiphyseal dysplasia |
| Q77.8 | Other osteochondrodysplasia with defects of growth of tubular bones and spine |
| Q77.9 | Osteochondrodysplasia with defects of growth of tubular bones and spine, unspecified |
| **Q78** | **Other osteochondrodysplasias** |
| Q78.0 | Osteogenesis imperfecta  Fragilitas ossium  Osteopsathyrosis |
| Q78.1 | Polyostotic fibrous dysplasia  Albright(-McCune) (-Sternberg) syndrome |
| Q78.2 | Osteopetrosis  Albers-Schönberg syndrome |
| Q78.3 | Progressive diaphyseal dysplasia  Camurati-Engelmann syndrome |
| Q78.4 | Enchondromatosis  Maffucci syndrome  Ollier disease |
| Q78.5 | Metaphyseal dysplasia  Pyle syndrome |
| Q78.6 | Multiple congenital exostoses  Diaphyseal aclasis |
| Q78.8 | Other specified osteochondrodysplasias  Osteopoikilosis |
| Q78.9 | Osteochondrodysplasia, unspecified  Chondrodystrophy (not otherwise specified)  Osteodystrophy (not otherwise specified) |

**References**

1. World Health Organization. International Statistical Classification of Diseases and Related Health Problems: tenth revision. 2nd edition. Geneva: World Health Organization. 2004.
